# Determinants of COVID-19 Vaccine Hesitancy: A Cross-Sectional Study on a Mexican Population Using an Online Questionnaire (COV-AHQ)

**DOI:** 10.3389/fpubh.2021.728690

**Published:** 2021-11-26

**Authors:** Juan Luis Delgado-Gallegos, Gerardo R. Padilla-Rivas, Erika Zúñiga-Violante, Gener Avilés-Rodríguez, Daniel Arellanos-Soto, Lilia Julieta Gastelum-Arias, Héctor Franco Villareal, María de los Ángeles Cosío-León, Gerardo Salvador Romo-Cardenas, María G. Moreno-Treviño, Jorge E. Moreno-Cuevas, Jose Francisco Islas

**Affiliations:** ^1^Departamento de Bioquímica y Medicina Molecular, Facultad de Medicina, Universidad Autónoma de Nuevo León, San Nicolás de los Garza, Mexico; ^2^Universidad de Montemorelos, Montemorelos, Mexico; ^3^Escuela de ciencias de la salud, Universidad Autónoma de Baja California, Mexicali, Mexico; ^4^Althian Clinical Research, Monterrey, Mexico; ^5^Dirección de investigación e innovación y posgrado Universidad Politécnica de Pachuca, Hidalgo, Mexico; ^6^Facultad de Ingeniería, Arquitectura y Diseño, Universidad Autónoma de Baja California, Mexicali, Mexico; ^7^Departamento de ciencias básicas, Universidad de Monterrey, San Pedro Garza García, Mexico

**Keywords:** COVID-19 vaccine hesitancy in Mexico, COVID-19 vaccine Latin-America, COVID-19 vaccine awareness, COVID-19 vaccination attitude, COVID-19 vaccine acceptance

## Abstract

Mexico has become one of the most highly affected countries by coronavirus disease 2019 (COVID-19) pandemic in Latin America. Therefore, efficient vaccination programs are needed to address COVID-19 pandemic. Although recent advances around the world have made it possible to develop vaccines in record time, there has been increasing fear and misinformation around the vaccines. Hence, understanding vaccine hesitancy is imperative for modeling successful vaccination strategies. In this study, we analyzed the attitude and perceptions toward COVID-19 vaccination, in a Mexican population (*n* = 1,512), using the proposed COVID-19 Vaccine Acceptance and Hesitancy Questionnaire (COV-AHQ) (Cronbach's alpha > 0.8), which evaluates a mild perception of danger and contamination with respect to COVID-19, a moderate perception of xenophobia generated throughout COVID-19 quarantine, fear of adverse effects of COVID-19 vaccination, and hesitancy of parent toward vaccination of children; furthermore, a section including sociodemographic variables was included. According to the results of this study, the statistical correlation analysis of the general vaccination posture seems to correlate significantly (*p* < 0.05) with a mild perception of danger and contamination with respect to COVID-19, a moderate perception of xenophobia generated throughout COVID-19 quarantine, hesitancy of parent toward vaccination of children, willingness to get COVID-19 vaccine, previous influenza vaccination, perception of the vaccine that could help the economy of country, occupation, gender, age, and participants actively researching COVID-19 vaccine information. An in-depth analysis assisted by binary logistic regression concluded that the young adult population around ages 18–34 years are the most likely to get vaccinated. This posture seems to be highly influenced by a mild perception of danger and contamination with respect to COVID-19, a moderate perception of xenophobia generated throughout COVID-19 quarantine, fear of adverse effects of COVID-19 vaccination, and hesitancy of parents toward vaccination of children. While their own personal religious beliefs and economic status, the level of education does not seem to have an effect on the willingness to get vaccinated neither did having a previous COVID-19 diagnosis or even knowing someone with a positive COVID-19 diagnosis. Health authorities and policymakers could use the results of this study to aid in modeling vaccination programs and strategies and identify population groups with high vaccine hesitancy prevalence and assess significant public health issues.

## Introduction

The highly infectious severe acute respiratory syndrome coronavirus-2 (SARS-CoV-2), which gives rise to coronavirus disease 2019 (COVID-19) (1), has through its tenure infected over 106 million people worldwide, while claiming the lives of over 2.3 million (2). According to the John Hopkins Coronavirus Resource Center, the highest hit countries by total cases are the US, Brazil, and UK (top 5%), but if we rank by death rate, Mexico is placed among the top ranks (top 1%) with about 9% death rate or case-fatality ratio, closely followed by Peru, Panama, Brazil, Colombia, and Ecuador, all of which are Latin American countries, with similar or slightly lower case-fatality ratio (2, 3).

Interestingly, healthcare system of Mexico has been a top priority for the country, leading to excellent results even in times of political and financial hardships (4). Regrettably, due to many factors such as the rise in overall healthcare cost and the need to reallocate assets, over time healthcare system of Mexico became fragmented with ill-equipped and understaffed hospitals (5), a paramount problem when trying to combat the current epidemic. It is important to mention that Mexico is a country where more than a quarter of adults develop hypertension and cardiovascular disease and close to 15% have diabetes, as all of these comorbidities might aggravate the condition of a patient infected with COVID-19 (2, 6–9).

Recent technological advances have made possible the acceleration of COVID-19 vaccines design and production. Currently, Mexico is in the last stages of clinical protocol evaluations; hence, Mexico is awaiting the approval of several vaccines for their use in the population (1, 10). Despite successful mass vaccination scenarios recorded throughout history such as polio and smallpox (11, 12), distrust in newly developed vaccines has grown, as there are many myths and misinformation around them (11, 13), Therefore, all the vaccination strategies should take into consideration factors associated with acceptance of the population. “What if I do not want to get a shot?,” “Do I fear adverse effects?;” recently, a multinational survey showed that of 13,426 people from 19 countries, 28.5% reported that they would not apply the vaccine (14). Although side effects associated with vaccination are generally mild, for example, pain and/or bleeding at the application site and temporary general malaise, some serious side effects reported are Guillain-Barré syndrome, febrile seizures, intussusception, coagulopathies, or anaphylaxis (11). Despite measures to counteract mistrust and misinformation around vaccination, these efforts have been frequently ineffective (12). To overcome this, authorities need a proactive approach in order to strengthen vaccination culture, leading with accurate scientific information and emphasizing the importance of vaccines in preventing life-threatening diseases (11, 12). A study by Timmis et al. determined a set of potential attributes in pro of acceptance of applying the vaccine: (1) High incidence of cases prevented per year, (2) Cost-effectiveness, (3) Being disease of high mortality, and (4) Risk of morbidity and mortality (15). Therefore, it is important for the sanitary authorities to strengthen the messages about the positive benefits of taking the vaccine in the general population.

We made a questionnaire based on the Adapted Covid Stress Scales and Vaccine Hesitancy Scales to determine how and which factors play crucial roles in the overall hesitancy of taking the vaccine (16–19). In this study, we look at four major sections as they related to a mild perception of danger and contamination with respect to COVID-19, a moderate perception of xenophobia generated throughout COVID-19 quarantine, fear of adverse effects of COVID-19 vaccination, and hesitancy of parents toward vaccination of children. We also looked at the sociodemographic profile to determine how all these variables correlate. The overall purpose is to understand the levels of acceptance of vaccination against COVID-19 in Mexico and how different variables affect the perception of this event.

## Materials and Methods

This study proposes the application of COVID-19 Vaccine Acceptance and Hesitancy Questionnaire (COV-AHQ), designed by the authors based on the Adapted COVID-19 Stress Scale (ACSS) (16, 17) and the Vaccine Hesitancy Scale (VHS) (18). We wrote our questionnaire using MS Forms (Microsoft Corporation, Redwood, Washington, United States) and applied it remotely through a web link, as seen in Table 1. We distributed this test to the general population in Mexico through electronic means such as social media groups: Facebook, Twitter, Reddit, and directed e-mail. We applied the questionnaire throughout the period from December 2020 to February 2021 before the beginning of the vaccination program in Mexico (20).

**Table 1:** COVID-19 Vaccine Acceptance and Hesitancy Questionnaire (COV-AHQ).

**Initial questions**
1. Do you want to participate in the questionnaire?^*^
2. What is your occupation?
3. What is your gender?
4. What is your age?
5. What is your academic degree?
6. State where I currently live
7. How many people live in your household, including you?
8. How many rooms do you have in your household? (including kitchen and living room)
9. Do you suffer from any risky disease?
10. Do you have a religion?
Section 1 (Danger and contamination)
11. I am worried about getting the virus
12. I am worried about being asymptomatic and infecting my loved ones.
13. I am concerned that social distancing is not enough to keep me safe from the virus
14. I am worried that the vaccine runs out before I get vaccinated
**Section 2 (Xenophobia)**
15. I am concerned that people out of state are spreading the virus.
16. I am concerned that people I know who live outside of my state may have the virus.
17. I am concerned about encountering people out of state because they may have the virus.
18. I am concerned to hang out with people that does not want to get vaccinated
**Section 3 (Fear of vaccination's adverse effects)**
19. I am concerned to get any type of vaccine
20. I am worried to develop an adverse reaction related to the COVID-19 vaccine
21. I am worried that the vaccine against COVID-19, gets me or my relative's sick
22. I am worried about getting vaccinated because I already had COVID-19
**Section 4 (Parent's hesitancy toward children vaccination)**
23. I consider that getting my child vaccinated it is important for the health of others in my community
24. I consider that the new vaccines against COVID have more risk than others (e.g., influenza)
25. I consider that getting my child vaccinated is a good protective measure
26. I am concerned about my child developing an adverse effect related to the COVID vaccination
**COVID questions**
27. Have you been diagnosed with COVID-19 previously?
28. Do you know someone who has been diagnosed with COVID-19?
29. What is your posture toward vaccination?
30. Did you get vaccinated with seasonal influenza in the year 2020 or 2021?
31. Do you consider that the COVID-19 vaccine can improve the current social, economic and/or health situation?
32. Are you willing to get the COVID-19 vaccine?
33. Have you searched for information regarding the COVID-19 vaccine?
34. What types of COVID-19 vaccine do you know?
**Final questions for future follow-up**
35. Would you be interested in taking part in a questionnaire to monitor your mental health in the future?
36. We appreciate your interest and we ask that you please leave us an email address

* *Consent to participate*.

In order to calculate the sample size needed, we used the classical method by Lwanga and Lemershow (1991) for a finite population. For Mexico, it accounts for using a total population for 130 million as input (21). Additionally, we used a *z*-value of 1.96 (confidence level of 97.5%) and an expected *p*-value of 0.04 (expected percentage of cases) based on official data. The resulting value was an expected *n* of 1475.15.

All the subjects acknowledged being >18 years old and gave their consent for inclusion before participating in this study. We used a Likert scale format with increasing point values to further classify according to scores (16, 22). We calculated all the statistical correlation analyses using IBM-SPSS Statistics for Windows, version 23.0 (IBM Corporation, Armonk, New York, USA) with the Pearson's chi-squared test and an R ratio of 0.05. We calculated the frequency of answers in relation to categories and other sociodemographic variables. We then correlated the answers to the number of points in each section. Section 1 evaluated a mild perception of danger and contamination with respect to COVID-19, Section 2 evaluated a moderate perception of xenophobia generated throughout COVID-19 quarantine, and Section 3 evaluated fear of adverse effects of COVID-19 vaccination. Finally, Section 4 evaluated hesitancy of parents toward vaccination of children (18). We classified the resulting scores for Sections 1 to 4 and their ranges according to the following scales: absent 0 to 4, mild 5 to 8, moderate 9 to 12, and severe 13 to 16. These classifications evaluate the impact each individual section has on the daily life of participant. Where on one end, absent describes a negligible effect of the section on the participant, to severe where the section highly influences the daily decisions of participants. Other studied items were respect to different sociodemographic and vaccination variables such as occupation, gender, age, practice of religion, education level, total of habitants in household, total number of rooms in household, disease/comorbidity, knowing someone positive for COVID-19, attitude toward vaccination, previous influenza vaccination for 2020–2021 season, consideration of COVID-19 vaccine as a mean of turning a positive tide on the current socioeconomic situation of the country, willingness to receive COVID-19 vaccination, participants actively researching COVID-19 vaccine information, and their willingness to continue participating in follow-up questionnaires. To ensure integrity of the questionnaire, we consulted a group of experts to perform and evaluate pertinence and clarity tests. The calculated Cronbach's alpha (>0.8) represented good internal consistency. To identify the factors, which would have the most impact on the overall perception of acceptance among the participants, we used binary logistic regression analysis. This model was based on using the demographic factors and the studied sections as a whole (a mild perception of danger and contamination with respect to COVID-19, a moderate perception of xenophobia generated throughout COVID-19 quarantine, fear of adverse effects of COVID-19 vaccination, and hesitancy of parents toward vaccination of children associated with willingness) to correlate them to the willingness to get vaccinated. We then introduced these selected factors and sections into a backward stepwise (likelihood ratio) method. Finally, we used to quantify the associations between factors and sections and willingness to vaccine unstandardized regression coefficients (ß) and odds ratios (ORs) and their 95% CIs.

## Results

Given the extent COVID-19 pandemic has affected the country (2, 17, 23) and how recent advances in technology have moved forward interconnectivity (23–25), we opted to use electronic means to implement our questionnaire, as this becomes both a permissible way to distribute to the general population and to follow preventive measures such as social distancing. As earlier stated, we used direct email invitation and posting links to the questionnaire in popular social media platforms used by the public. Obtaining 1,512 participants, from which 1,481 (97.9%) participants accepted to participate in this study, while 31 (2.1%) participants declined. We should note that throughout the questionnaire, it did not require participants to answer all the questions to advance. We present the general sociodemographic information for all the consenting participants in Table 2. Out of the initial sociodemographic assessment, the most representative results showed the following information: for gender, there was a slight majority of females (57.9%). The most frequent age range reported was 25 to 34 years (28.6%). The most common occupation status was students (20.2%). sixty-eighth percentage of participants reported practicing a religion, while 55% of participants reported having a bachelor's degree. Interestingly, the most common household occupancy was of four people (27.2%) and the most regular reported house size was of >4 rooms (61.7%). As expected, 30.3% of the participants reported having at least one comorbidity obesity being the highest in frequency (61.1%), followed by cardiovascular diseases (27.6%) and diabetes (14.2%). Forty-Six percentage of participants reported having children. Meanwhile, 82.3% of participants reported not having a previous diagnosis of COVID-19, while 95.7% of participants reported knowing at least someone with COVID-19 diagnosis. Remarkably, 80.2% of participants reported a positive posture toward vaccination. An interesting observation was that 48.7% of participants reported their shoot for influenza in the 2020–2021 season. Unsurprisingly, 86.2% of participants agreed that COVID-19 vaccine will help to advance the economic situation of the country, while 87.8% of participants reported a willingness to get the vaccine against COVID-19. Finally, 73.1% of participants reported actively looking for COVID-19 vaccine-related information.

**Table 2:** Social demographic profiles of participants.

	***n***	**%**
**Total participants**	1,512	100.00%
Accept	1,481	97.90%
Decline	31	2.10%
**Gender**		
Male	568	38.40%
Female	857	57.90%
Other	2	0.10%
No answer	54	3.60%
**Age (range)**		
18 to 24 years	295	19.90%
25 to 34 years	423	28.60%
35 to 44 years	416	28.10%
45 to 54 years	202	13.60%
55 to 64 years	102	6.90%
>65 years	35	2.40%
No answer	8	0.50%
**Employment**		
Unemployed	70	4.70%
Student	299	20.20%
Health professional	175	11.80%
Essential worker	245	16.50%
Non-essential worker	183	12.40%
Commerce	67	4.50%
Academic professional	205	13.80%
Other	229	15.50%
No answer	8	0.50%
**Religion (practice)**		
Yes	1,013	67.00%
No	441	29.20%
No answer	27	1.80%
**Education level (degree)**		
Elementary	3	0.20%
Jr Highschool	18	1.20%
Highschool	224	14.80%
Bachelors	816	54.00%
Graduate	402	26.60%
Not apply	13	0.90%
No answer	4	0.30%
**Household occupants**		
1	98	6.50%
2	276	18.30%
3	313	20.70%
4	412	27.20%
>4	347	22.90%
No answer	35	2.30%
**Rooms**		
1	18	1.20%
2	70	4.60%
3	164	10.80%
4	258	17.10%
>4	933	61.70%
No answer	38	2.50%
**Comorbidities**		
Yes, to having at least one comorbidity	449	31.80%
No	962	63.60%
No answer	70	4.60%
**Reported comorbidities**		
CVD	124	21.90%
Diabetes	64	11.30%
Pulmonary disease	53	9.40%
Cancer	5	0.90%
HIV	3	0.50%
Obesity	278	49.10%
**Having children**		
Yes	682	45.10%
No	773	51.10%
No answer	26	1.70%
**Having a previous COVID-19 diagnosis**		
Yes	230	15.20%
No	1,219	80.60%
No answer	32	2.10%
**Knowing someone with COVID-19**		
Yes	1,417	93.70%
No	32	2.10%
No answer	33	2.20%
**Attitude toward vaccination**		
Agree	1,188	78.60%
Neutral	218	14.40%
Disagree	43	2.80%
No answer	32	2.10%
**Influenza vaccine during the period of 2020–2021**		
Vaccinated	736	48.70%
No vaccinated	715	47.30%
No answer	30	2.00%
**The COVID-19 vaccine will enhance the economic situation**		
Agree	1,303	86.20%
Disagree	144	9.50%
No answer	35	2.30%
**Willingness to get COVID-19 vaccinated**		
Yes	1,328	87.80%
No	120	7.90%
No answer	33	2.20%
**Participants actively researching COVID-19 vaccine information**		
Yes	1,105	73.10%
No	342	22.60%
No answer	35	2.30%

**Figure 1:**
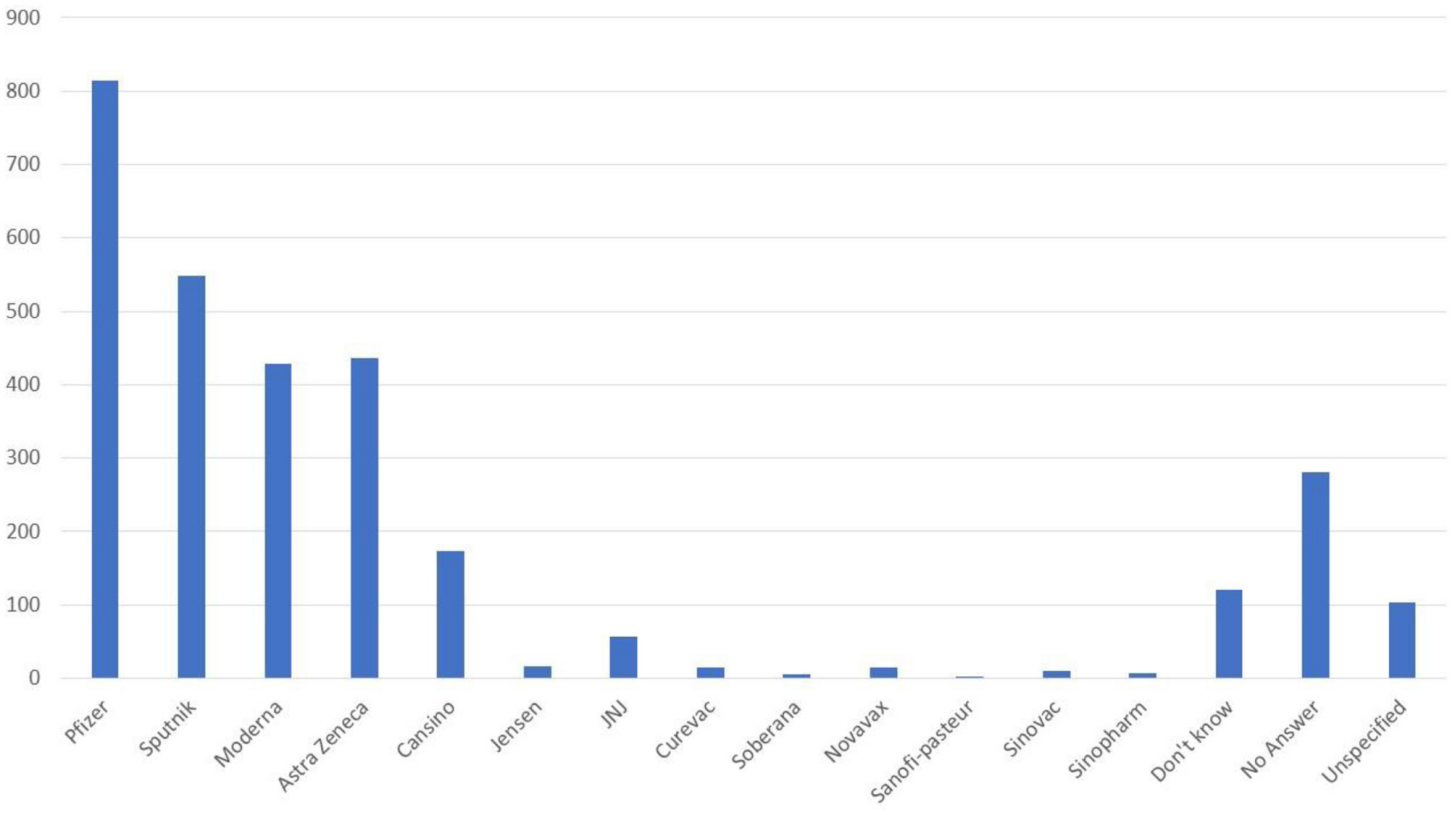
Frequency counts as the most researched coronavirus disease 2019 (COVID-19) vaccines brands.

Next, we evaluated the four sections of the questionnaire and correlated them with all the variables studied (sociodemographic and COVID-19-related questions) and results are seen in Table 3. From these results, the most relevant classifications were: occupation showed statistical relevance to Section 2 (moderate), Section 3 (absent), and Section 4 (absent) all with a *p* < 0.001. Gender showed statistical relevance to Section 3 (absent) and Section 4 (absent) all with a *p* < 0.001. Age showed statistical relevance to Section 1 (mild) (*p* < 0.005), Section 2 (moderate) (*p* < 0.001), Section 3 (absent) (*p* < 0.010), and Section 4 (absent) (*p* < 0.001). For religion, Section 3 (absent) and Section 4 (absent) showed statistical relevance with a *p* < 0.001. Education level showed statistical relevance to Section 3 (absent) (*p* < 0.001). Household occupants showed statistical relevance to Section 4 (absent) (*p* < 0.001). Meanwhile, the total number of rooms only showed relevance to Section 4 (absent) (*p* < 0.002). Interestingly, participants having at least only one comorbidity showed relevance to Section 1 (mild) (*p* < 0.016) and Section 4 (absent) (*p* < 0.001). A previous COVID-19 diagnosis was statistically relevant only to Section 3 (absent) (*p* < 0.001). Knowing someone with COVID-19 diagnosis showed statistical relevance to Section 4 (absent) (*p* < 0.001). General posture toward vaccination applied to Section 1 (mild), Section 2 (moderate), Section 3 (absent), and Section 4 (moderate) all with a *p* < 0.001. Getting influenza vaccination for the 2020–2021 season was statistically relevant to Section 1 (mild) (*p* < 0.001), Section 2 (moderate) (*p* < 0.007), and Section 3 (absent) (*p* < 0.001). Considering that COVID-19 vaccine will help to boost the economic situation of country, it showed statistical relevance to Section 1 (mild), Section 2 (moderate), Section 3 (absent), and Section 4 (moderate) all with a *p* < 0.001. Willingness to get COVID-19 vaccinated showed statistical relevance to Section 1 (mild), Section 2 (moderate), Section 3 (absent), and Section 4 (moderate) all with a *p* < 0.001. Participants actively researching COVID-19 vaccine information was statistically relevant to Section 1 (mild) (*p* < 0.011), Section 3 (absent) (*p* < 0.001), and Section 4 (moderate) (*p* < 0.016). A previous COVID-19 diagnosis was only relevant to Section 3 (absent) (*p* < 0.001). We further correlated all the variables studied among themselves (Table 4). We asked if the participant was actively searching for information about the vaccines. Most of the research conducted was about Pfizer, Sputnik V, Moderna, and AstraZeneca vaccines, as seen in Figure 1. Finally, using the SPSS^®^ software, we did binary logistic regression analysis and results are shown in Table 5. The algorithm plucked in a stepwise manner and demographic variables did not influence the outcome of willingness to get vaccinated. Results showed that in the age groups from 18 to 24 years (*p* < 0.001, OR: 8.36) and in the age groups from 25 to 34 years (*p* < 0.019, OR: 2.36), the only demographic variables influencing the willingness to get vaccinated. Within the four studied sections, particular categories participated in the willingness to get vaccinated: a mild perception of danger and contamination with respect to COVID-19: mild (*p* < 0.001, OR: 4.60), moderate (*p* < 0.001, OR: 24.50), and severe (*p* < 0.001, OR: 48.41); a moderate perception of xenophobia generated throughout COVID-19 quarantine: absent (*p* < 0.001, OR: 0.16) and mild (*p* < 0.045, OR: 0.52); fear of adverse effects of COVID-19 vaccination: absent (*p* < 0.001, OR: 141.70), mild (*p* < 0.001, OR: 23.39), and moderate (*p* < 0.018, OR: 3.40); and hesitancy of parents toward vaccination of children: absent (*p* < 0.001, OR: 0.16), mild (*p* < 0.001, OR: 0.07), and moderate (*p* < 0.004, OR: 0.20).

**Table 3:** Statistical correlation (*p*-values) for social demographics and coronavirus disease 2019 (COVID-19)-related questions and sections (1–4).

**Social demographics and vaccination variables questions**	**Danger and contamination (Section 1)**	**Xenophobia (Section 2)**	**Fear of vaccinations adverse effects (Section 3)**	**Parent's hesitancy toward children's vaccination (Section 4)**
Occupation	*p <* 0.110	**p < 0.001**	***p <*** **0.001**	*p <* 0.089
Gender	*p <* 0.431	*p <* 0.432	***p <*** **0.001**	*p <* 0.366
Age	***p <*** **0.005**	***p <*** **0.001**	***p <*** **0.01**	***p <*** **0.036**
Religion (practice)	*p <* 0.144	*p <* 0.140	***p <*** **0.001**	*p <* 0.274
Education level	*p <* 0.108	*p <* 0.251	***p <*** **0.001**	*p <* 0.065
Total number of occupants	*p <* 0.870	*p <* 0.131	*p <* 0.198	***p <*** **0.023**
Total number of rooms	*p <* 0.182	*p <* 0.214	*p <* 0.447	*p <* 0.272
Do you know someone positive for COVID-19	*p <* 0.932	*p <* 0.196	*p <* 0.097	***p <*** **0.001**
General posture on vaccination	***p <*** **0.001**	***p <*** **0.001**	***p <*** **0.001**	***p <*** **0.001**
Influenza vaccination for 2020-2021 season	***p <*** **0.001**	***p <*** **0.007**	***p <*** **0.001**	*p <* 0.371
Consideration of a COVID-19 vaccine as means of turning a positive tide on the current social economical situation of the country	***p <*** **0.001**	***p <*** **0.001**	***p <*** **0.001**	***p <*** **0.001**
Willingness to receive the COVID-19 vaccine	***p <*** **0.001**	***p <*** **0.001**	***p <*** **0.001**	***p <*** **0.001**
Research of the different COVID-19 vaccines by paper or electronic means	***p <*** **0.011**	*p <* 0.054	***p <*** **0.001**	***p <*** **0.018**
Having a previous COVID-19 diagnosis	*p <* 0.743	*p <* 0.774	***p <*** **0.001**	*p <* 0.787

*Bold values are Significative P value*.

**Table 4:** Statistical correlation (*p*-values) among all the social demographics and COVID-19-related questions.

**Social Demographics and vaccination variables questions**	**1. Occupation**	**2. Gender**	**3. Age**	**4. Religion (practice)**	**5. Education level**	**6. Total number of occupants**	**7. Total number of rooms**	**8. Do you know someone positive for COVID-19**	**9. General posture onvaccination**	**10. Influenza vaccination for 2020–2021 season**	**11. Consideration of a COVID-19 vaccine as means of turning a positive tide on the current social economical situation of the country**	**12. Willingness to receive the COVID-19 vaccine**	**13. Research of the different COVID-19 vaccines by paper or electronic means**	**14. Having a previous COVID-19 diagnosis**
1		**<0.001**	**<0.001**	**<0.001**	**<0.001**	**<0.001**	**<0.001**	0.219	**<0.001**	**<0.001**	**0.029**	**<0.001**	**<0.001**	**0.004**
2	**<0.001**		**<0.001**	**<0.001**	**<0.001**	0.131	**<0.001**	0.956	**0.024**	**0.004**	0.161	**0.005**	**<0.001**	0.539
3	**<0.001**	**<0.001**	1	**<0.001**	**<0.001**	**<0.001**	0.138	0.051	**0.014**	**<0.001**	0.093	**<0.001**	0.352	0.233
4	**<0.001**	**<0.001**	**<0.001**		0.761	**0.003**	0.45	0.637	0.496	**0.027**	0.52	0.543	0.442	0.133
5	**<0.001**	**<0.001**	**<0.001**	0.761		**<0.001**	**0.002**	0.464	**0.017**	**0.013**	0.499	0.15	**<0.001**	0.489
6	**<0.001**	0.131	**<0.001**	**0.003**	**<0.001**		**<0.001**	0.928	0.996	0.426	0.826	0.696	0.339	**0.043**
7	**<0.001**	**<0.001**	0.138	0.45	**0.002**	**<0.001**		0.075	**0.009**	**0.041**	0.063	**0.04**	**<0.001**	0.073
8	0.219	0.956	0.051	0.637	0.464	0.928	0.075		0.537	0.895	0.092	0.287	**<0.001**	**0.015**
9	**<0.001**	**0.024**	**0.014**	0.496	**0.017**	0.996	**0.009**	0.537	2	**<0.001**	**<0.001**	**<0.001**	**<0.001**	0.197
10	**<0.001**	**0.004**	**<0.001**	**0.027**	**0.013**	0.426	0.041	0.895	**<0.001**	3	**<0.001**	**<0.001**	**<0.001**	0.114
11	**0.029**	0.161	0.093	0.52	0.499	0.826	0.063	0.092	**<0.001**	**<0.001**	4	**<0.001**	**<0.001**	0.143
12	**<0.001**	**0.005**	**<0.001**	0.543	0.15	0.696	**0.04**	0.287	**<0.001**	**<0.001**	**<0.001**	5	**<0.001**	0.728
13	**<0.001**	**<0.001**	0.352	0.442	**<0.001**	0.339	**<0.001**	**<0.001**	**<0.001**	**<0.001**	**<0.001**	**<0.001**	6	0.613
14	**0.004**	0.539	0.233	0.133	0.489	**0.043**	0.073	**0.015**	0.197	0.114	0.143	0.728	0.613	

*Similar numbers in both axes represent same variables. Bold values are Significative P value*.

**Table 5:** Results of binary logistic regression analysis on demographic factors and sections significantly associated with willingness to get vaccinated.

**Variables**		**B**	**Standard error**	**Wald**	**gl**	**(***p***-value)**	**OR**	**95% CI for OR**	
								**Lower**	**Upper**
Danger and contamination	Mild	1.53	0.29	26.88	1	**0.001**	4.6	2.583	8.19
	Moderate	3.2	0.5	41.33	1	**0.001**	24.5	9.24	64.967
	Severe	3.88	0.87	19.96	1	**0.001**	48.41	8.824	265.555
Xenophobia	Absent	−1.86	0.37	25.1	1	**0.001**	0.16	0.075	0.322
	Mild	−0.65	0.32	4.03	1	**0.045**	0.52	0.277	0.985
Fear of vaccination's adverse effects	Absent	4.95	0.59	71.41	1	**0.001**	141.7	44.914	447.035
	Mild	3.15	0.55	33.42	1	**0.001**	23.39	8.033	68.112
	Moderate	1.22	0.52	5.63	1	**0.018**	3.4	1.237	9.331
Parent's vaccination hesitancy in children	Absent	−1.84	0.57	10.46	1	**0.001**	0.16	0.052	0.484
	Mild	−2.66	0.61	19.08	1	**0.001**	0.07	0.021	0.231
	Moderate	−1.59	0.56	8.08	1	**0.004**	0.2	0.069	0.611
Aged Range	18 to 24 years	2.12	0.56	14.26	1	**0.001**	8.36	2.777	25.166
	25 to 34 years	0.86	0.36	5.54	1	**0.019**	2.36	1.154	4.815
	35 to 44 years	0.58	0.32	3.16	1	0.075	1.78	0.943	3.364
	55 to 64 years	0.84	0.47	3.17	1	0.075	2.31	0.919	5.828

*Model summary: −2 log likelihood = 472.980, Cox and Snell R square = 0.203, and Nagelkerke R square = 0.471. Bold values are Significative P value*.

## Discussion

The WHO stated in the last year that vaccinations effectively save millions of lives every year; they recognize that vaccination programs are “one of the world's most successful and cost-effective health interventions,” which prevent over 2 million deaths every year.

In Mexico, influenza vaccination has proven to be effective, albeit there are still several declared cases, it continues well controlled in the overall population (4, 26). Even now with COVID-19 pandemic, we registered that almost 50% of the surveyed population got vaccinated for influenza, even considering the potential risks of going to a health center amid COVID-19 pandemic (27). This seems to fair with what other researcher had observed back in 2020 when a survey by Forbes concluded that there was a massive increase in the demand for influenza vaccine, as this might protect against notable effects of COVID-19 (28). The results in this study suggest that the general population in Mexico has a good vaccination culture, as 78.6% agreed to have a positive posture toward vaccination. Occupations, such as essential workers, students, and healthcare professionals, showed to have a good affinity toward the idea of vaccination. A recent study showed that those involved in the care of patients with COVID-19 had higher levels of vaccination acceptance than those not directly attend patients with COVID-19 positive (29).

Over the course of the past year, the perception toward vaccination may have potentially changed. Currently, the mortality rate in Mexico is around 9% (2, 3) and 95.7% of participants admitted knowing someone diagnosed with COVID-19, while 15.5% of participants had a previous COVID-19 diagnosis. Therefore, a potential preventive measure such as a vaccine would ease at least partially the overall stress felt by the general population. It is easy to assume that a portion of the participants have had contact with a patient that passed away or that has been in a delicate state because of COVID-19. This idea of potential change toward positive views in vaccination is further supported by the results of the questionnaire sections evaluating the different aspects toward fear and contamination and xenophobia related to COVID-19 in Sections 1 and 2 of the questionnaire. Our binary logistic regression analysis further weighed in favor of Section 1 as the OR for moderate: 24.50 and severe: 48.41. Binary logistic regression analysis also showed that Section 3 had a highly important contribution as being absent had an OR of 141.70, with mild contributing with an OR of 23.39; this is interesting as precisely the fear of adverse effects of COVID-19 vaccination correlated with the willingness to get COVID-19 vaccinated. Over 49% of participants scored in the absent category. Thus, affecting the overall desire to get vaccinated as 87.8% of the population showed an interest to get COVID-19 vaccine and 73.1% of participants are actively looking for information with respect to COVID-19 vaccine. Nearly half of the participants (48.7%) got vaccinated against seasonal influenza during the 2020–2021 period; potentially, the rest might not have gotten the vaccine out of fear of getting infected when going to a healthcare center (27). However, a newly realized study among nursing professionals showed that COVID-19 vaccination intention directly relates to a previous influenza vaccination, where the most important associated factors were young age, confidence, and collective responsibility (30). By comparison, we can draw similarities with the general population in Mexico, where the influenza vaccination rate grew over 2019 above 300% (28), as there is an overall positive approval rate toward getting vaccinated. In our result section, we see several correlations among variables related to hesitancy and the four general sections, which evaluate the sense of a mild perception of danger and contamination with respect to COVID-19, a moderate perception of xenophobia generated throughout COVID-19 quarantine, fear of adverse effects of COVID-19 vaccination, and hesitancy of parents toward vaccination of children.

Researchers have recently described factors associated with a decline in vaccination such as exposure to other people while being in line to get vaccinated against COVID-19 as one of the major concerns, as individuals might get infected and possibly spread the virus (27). Also, concerns about safety and effectiveness of the vaccine are noted in the literature and doubts toward the vaccines approval derived from their rapid development, delayed side effects presentation, and misinformation about COVID-19 vaccination on social media (31). Religious beliefs pose interesting perspectives. On one hand, religion seems to have a protective measure against stress related to COVID-19 quarantine and in most cases, it does not correlate toward the application of the vaccine (32). Another variable studied was cohabitation, which did not show any correlations toward protective measures or toward a positive attitude about receiving the vaccine. Patients that have high risk of comorbidities show a more aggressive presentation of the disease. In this study, we found that having any level of education seems to have a positive attitude toward vaccination and less fear toward COVID-19 vaccine. Females showed a significant correlation between variables with respect to a positive attitude toward COVID-19 vaccination. In addition, females seem to show more fear toward a possible adverse reaction. A study by Larson et al. found that these same trends applying similar survey in 2016 (33).

Coronavirus disease 2019 has set an unprecedented stress on the economy of world, as of March 2020 prediction for each additional month of quarantine would cost 2.5–3% of gross domestic product (GDP) and have a high impact on several countries (34). Given the economic crisis that the quarantine has set in Mexico among all the sectors, 86.2% of participants agreed that the application of COVID-19 vaccine will enhance the economic situation. This perspective is an important posture for the overall sentiment within the country, as the positive perception of the vaccines assisting the economy, potentially helps people cope with the overall economic stress generated during the quarantine, and finally seeing a light at the end of the tunnel.

Because of COVID-19 quarantine and at the time of the social distancing restrictions, we applied the questionnaire throughout a digital platform (Microsoft Forms). We could consider this as a limitation as it is unsupervised in-person, particularly during the quarantine, remote evaluations provide a safe alternative, albeit relying on the inclination of participant to answer. Currently, online surveys have been used by several groups, particularly in COVID-19 pandemic, to gather public or particular selected group information (17, 23, 35–37). As stated elsewhere in the manuscript, we distributed the questionnaire either by direct email invitation (we further asked participants to send to other colleagues or acquaintances the link to the questionnaire) or by popular social media platforms. However, this poses the problem of not being able to know the number of potential subjects to whom the questionnaire was available, hence not being able to calculate a participation rate. In addition, full completion of the questionnaire of participant was optional, as we believe that this might deter some from continuing. Our group distributes well among age groups, gender, types of employments, and other social demographic aspects as seen in Table 2.

## Conclusion

Even though COVID-19 vaccine has been associated with much uncertainty and misinformation around it, slowly but surely it is helping the world get through the quarantine. The development of new vaccines might save thousands of lives and eventually offset the balance of case-fatality ratio. Studies determining the factors required for acceptance of the vaccine or other important treatments are of the utmost importance, when trying to understand the behavioral patterns of a population, as many social and cultural factors can play crucial roles in the level of success of a preventive campaign such as that of a vaccination program.

In this study, we analyzed the attitude and perceptions toward vaccination, using the proposed COV-AHQ (with the calculated Cronbach's alpha >0.8). According to the binary logistics correlation analysis, the general vaccination posture seems to correlate significantly (*p* < 0.05) with a mild perception of danger and contamination with respect to COVID-19, a moderate perception of xenophobia generated throughout COVID-19 quarantine, hesitancy of parents toward vaccination of children, willingness to get COVID-19 vaccine, previous influenza vaccination, perception of the vaccine that could help the economy of country, occupation, gender, age, and participants actively researching COVID-19 vaccine information. A more in-depth analysis assisted by binary logistic regression analysis concluded that the young adult population around 18–34 years are the most likely to get vaccinated. This posture seems to be highly influenced by a mild perception of danger and contamination with respect to COVID-19, a moderate perception of xenophobia generated throughout COVID-19 quarantine, fear of adverse effects of COVID-19 vaccination, and hesitancy of parents toward vaccination of children. Their own personal religious beliefs, economic status (indirectly measured by household occupancy and total number of rooms), and the level of education do not seem to have an effect and having a previous COVID-19 diagnosis or even knowing someone with a positive COVID-19 diagnosis.

Given that young adults are the motor of the workforce in the country, actions to help with vaccination efforts should include flexible scheduling in vaccination campaign to address availability of different work shifts. Other positive actions that can be taken are enhancing company policies and measures toward employee vaccination such as providing transport for employees to vaccination sites or pay leave for vaccination purposes. Ideally, authorities should incentivize positive and proactive information campaigns directed at this population.

Current efforts by the government to vaccinate the population are being focused initially on at-risk groups, followed by a staggered vaccination divided in age groups. Our recommendation after these initial efforts to provide vaccination the entire population would be now to focus on a more community-based accessibility including local pharmacies and medical offices/hospitals as potential vaccination sites. In this second effort, we can expect a reduction in wait times, travel and expenses, exposure to harsh climate, and other inconveniences, making it more comfortable and accessible for the rest of the population to get vaccinated.

## Data Availability Statement

The raw data supporting the conclusions of this article will be made available by the authors, without undue reservation.

## Ethics Statement

The studies involving human participants were reviewed and approved by Comite de Etica, Hospital La Misión, Monterrey Nuevo León. The patients/participants provided their written informed consent to participate in this study.

## Author Contributions

JD-G and JI conceptualized and supervised the study, contributed to the overall design of the survey experiment, analysis and interpretation of the data, and wrote the first draft of the manuscript. GP-R, GR-C, HF, MC-L, and MM-T contributed to the overall design of the survey experiment, designed the discrete choice experiment, and contributed to the analysis and statistic interpretation of the data. EZ-V, GA-R, DA-S, LG-A, and JM-C contributed to the discussion of public health implications and helped to shape the overall interpretation. All the authors had access to all the data in this study and had final responsibility for the decision to submit for publication.

## Conflict of Interest

The authors declare that the research was conducted in the absence of any commercial or financial relationships that could be construed as a potential conflict of interest.

## Publisher's Note

All claims expressed in this article are solely those of the authors and do not necessarily represent those of their affiliated organizations, or those of the publisher, the editors and the reviewers. Any product that may be evaluated in this article, or claim that may be made by its manufacturer, is not guaranteed or endorsed by the publisher.
